# Haploidentical donor is preferred over matched sibling donor for pre-transplantation MRD positive ALL: a phase 3 genetically randomized study

**DOI:** 10.1186/s13045-020-00860-y

**Published:** 2020-03-30

**Authors:** Ying-Jun Chang, Yu Wang, Lan-Ping Xu, Xiao-Hui Zhang, Huan Chen, Yu-Hong Chen, Feng-Rong Wang, Wei-Han, Yu-Qian Sun, Chen-Hua Yan, Fei-Fei Tang, Xiao-Dong Mo, Yan-Rong Liu, Kai-Yan Liu, Xiao-Jun Huang

**Affiliations:** 1 Peking University People’s Hospital & Peking University Institute of Hematology, National Clinical Research Center for Hematologic Disease, Beijing Key Laboratory of Hematopoietic Stem Cell Transplantation, No. 11 South Street of Xizhimen, Xicheng District, Beijing, 100044 People’s Republic of China; 2 Peking-Tsinghua Center for Life Sciences, Beijing, 100871 China; 3 Research Unit of Key Technique for Diagnosis and Treatments of Hematologic Malignancies, Chinese Academy of Medical Sciences, Beijing, 2019RU029 China

**Keywords:** Haploidentical donor transplantation, Acute lymphoblastic leukemia, Matched sibling donor transplantation, Measurable residual disease, Donor selection

## Abstract

**Background:**

Previous reports suggest a benefit associated with haploidentical donor transplantation (HIDT) compared to matched sibling donor transplantation (MSDT) in certain contexts, and the choice of optimal candidates warrants further investigation.

**Methods:**

We designed a prospective genetically randomized study to evaluate donor options between acute lymphoblastic leukemia (ALL) patients positive for measurable residual disease (MRD) pre-transplantation who underwent HIDT (*n* = 169) or MSDT (*n* = 39).

**Results:**

The cumulative incidence of positive MRD post-transplantation was 26% (95% CI, 19–33%) and 44% (95% CI, 28–60%) for HIDT and MSDT, respectively (*P* = 0.043). Compared to the HIDT cohort, the MSDT cohort had a higher 3-year cumulative incidence of relapse (CIR; 47%, 95% CI, 31–63% vs. 23%, 95% CI, 17–29%; *P* = 0.006) and lower 3-year probability of leukemia-free survival (LFS; 43%, 95% CI, 27–59% vs. 65%, 95% CI, 58–72%; *P* = 0.023) and overall survival (OS; 46%, 95% CI, 30–62% vs. 68%, 95% CI, 61–75%; *P* = 0.039), without a difference in non-relapse-mortality (10%, 95% CI, 1–19% vs. 11%, 95% CI, 6–16%; *P* = 0.845). Multivariate analysis showed that HIDT is associated with a low CIR (HR = 0.364; 95% CI, 0.202–0.655; *P* = 0.001) and better LFS (HR = 0.414; 95% CI, 0.246–0.695; *P* = 0.001) and OS (HR = 0.380; 95% CI, 0.220–0.656; *P* = 0.001).

**Conclusions:**

HIDT is better than MSDT in view of favorable anti-leukemia activity for patients with pre-transplantation MRD positive ALL. The current study paves the way to determine that haploidentical donors are the preferred choice regardless of available matched sibling donors in a subgroup population.

**Trial registration:**

ClinicalTrials.gov Identifier: NCT02185261. Registered July 9, 2014. https://clinicaltrials.gov/ct2/show/NCT02185261?term=NCT02185261&draw=2&rank=1.

## Background

Currently, haploidentical donors have been an alternative source for allo-stem cell transplantation (SCT) for patients that require transplantation but have no related or unrelated donors with matching human leukocyte antigen (HLA) [1–5]. With the increasingly used haploidentical SCT (HIDT), HLA-identical sibling donors remain the first choice, though a number of studies have shown that treating patients with acute myeloid leukemia (AML) and acute lymphoblastic leukemia (ALL) using haploidentical donors (HIDs) could achieve comparable outcomes to those who undergoing HLA-matched sibling donor transplantation (MSDT) [3, 6, 7]. On the other hand, using haploidentical transplants, the graft-versus-leukemia (GVL) effect may be stronger, as mismatches for HLA antigens on leukemic cells would provide allo-immune targets [4, 5, 8–13]. A recent large European Society for Blood and Marrow Transplantation (EBMT) study indicated that HIDT has a lower incidence of relapse than MSDT for low-risk (HR = 0.83, *P* = 0.011) and intermediate-risk (HR = 0.85, *P* = 0.033) hematological malignancies [5]. The better relapse rate with HIDT compared to MSDT has also been observed in patients with lymphomas [8, 9]. Our previous studies showed that HIDT is superior to MSDT in terms of a lower relapse rate for patients with high-risk leukemia [11]. Furthermore, for older patients with acute leukemia, offspring donors not only result in lower non-relapse mortality (NRM), but also tended to be associated with a lower risk of relapse than MSDT [12]. Although these reports have effectively proven the potential superiority of HIDT to MSDT in the context of relapse risk in treating patients with some specified subgroups of hematological malignancies [4, 5, 8–12], they cannot inform decision-making in choosing one donor type over another for a specific patient due to the retrospective nature of the studies or highly diverse populations and various transplant regimens [4, 5, 8–13].

Apart from heterogeneous disease type, the variations in comparative outcomes between HIDT and MSDT could also be related to differences in disease status at the time of allo-SCT (i.e., less advanced disease or minimal residual disease [MRD]) [2, 4, 6, 13–17]. Therefore, studies have been performed in more homogenous groups [10]. Our group recently reported that, for AML patients with pre-transplantation MDR positivity (pre-HSCT MRDpos), HIDT could achieve a significantly lower cumulative incidence of relapse (CIR) and better survival than those who underwent MSDT [10]. Although this study included a prospective cohort with an homogenous population, there are some limitations with this prospective subgroup [10]. First, the sample size for pre-HSCT MRDpos (*n* = 76) was not large enough to reach reliable statistic power. Second, although the percentages of preemptive donor lymphocyte infusion (DLI) for post-HSCT MRD were described, there was neither a direct comparison of the incidence of post-transplantation MRD positivity (post-HSCT MRDpos) between donor sources nor a comparison of the proportion of interventions for post-HSCT MRDpos [10]. Considering these limitations [10], prospective comparative studies with enough power and more solid evidence of HIDT being better at eradiating leukemia cells are needed to challenge the traditional donor hierarchy of matching sibling donors (MSDs) being the first choice [14, 15].

For patients with ALL, a more recent EBMT study revealed that HIDT can obscure the negative effects of pre-HSCT MRDpos before transplantation in a subgroup analysis (CIR, 29% vs. 26% and leukemia-free survival, LFS, 50% vs. 50% for pre-HSCT MRDpos and pre-HSCT MRDneg, respectively) [16]. Though all of these findings [5, 8–11, 13] suggested a benefit associated with HIDT in certain contexts, the choice of an optimal candidate in terms of a stronger GVL effect warrants further investigation. Therefore, we designed a prospective genetically randomized study to evaluate donor options by comparing the endpoints related to disease control between ALL patients with pre-HSCT MRDpos who underwent HIDT and those who received MSDT. In this study with an homogenous population and unified transplant regimen, we provide convincing evidence that HIDT is favorable over MSDT in certain groups of patients, possibly by exerting a stronger GVL effect. Our results demonstrate that HIDT is associated with a lower incidence of post-HSCT MRDpos, lower CIR, and superior survival compared to MSDT. Our findings could have a major impact on donor selection regardless of available MSDs [14, 15].

## Methods

### Study design and patients

**Fig. 1:**
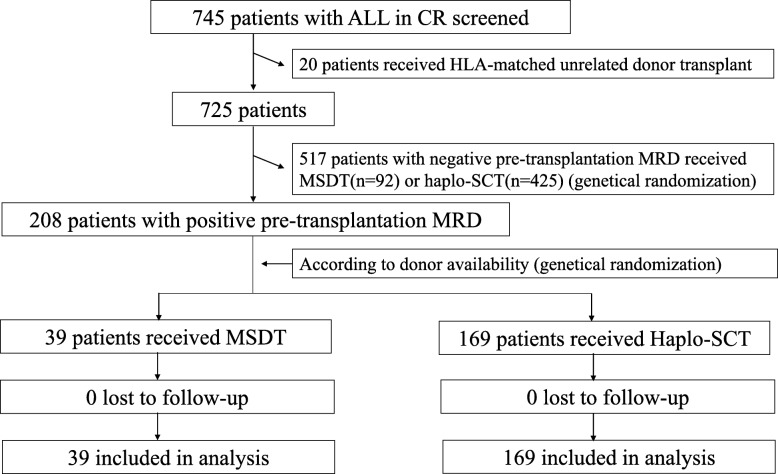
CONSORT (the Consolidated Standards of Reporting Trials) diagram. Abbreviations: ALL, acute lymphoblastic leukemia; CR, complete remission; HLA, human leukocyte antigen; MRD, measurable residual disease; MSDT, human leukocyte antigen-matched sibling donor transplantation; Haplo-SCT, haploidentical stem cell transplantation

This was a prospective cohort sub-study of a parent trial performed at Peking University Institute of Hematology (NCT02185261). Patients were assigned to groups transplanted with HIDT or MSDT based on donor availability (genetical randomization). Enrollment began in July 2014 and ended in February 2018. MSDT (6/6 matching HLA-A, B, and DR loci) was the first choice for allo-HSCT [14, 15]. If an HLA-matched sibling donor was unavailable, subjects without a suitable closely HLA-matched unrelated donor (> 8 of 10 matching HLA-A, B, C, DR, and DQ loci and > 5 of 6 matching HLA-A, B, and DR loci) after two cycles of consolidation were eligible for HLA-haplotype transplantation. For this comparative analysis to arrive at comparable patient cohorts that received transplants during the same time period, we excluded patients who underwent unrelated donor (URD) SCT (*n* = 20, Fig. 1). Patients who met the following criteria were included: age 3–65 years and ALL in complete remission (CR) with pre-HSCT MRDpos. Exclusion criteria were severe heart, kidney, or liver disease, a prior transplant, and hypersensitivity to rabbit anti-thymocyte globulin (ATG) if a haploidentical donor was available. The diagnosis of ALL was based on the NCCN criteria [17].

The MRD status was assessed in all patients in morphologic CR at transplant using validated methods (multiparametric flow cytometry for all patients, reverse transcriptase quantitative polymerase chain reaction for Philadelphia chromosome (PH) positive ALL, see below “detection of MRD”). As mentioned above, patients generally receive allo-HSCT after two cycles of consolidation and within 2 weeks after MRD assessment. Routine MRD monitoring was performed at 1, 2, 3, 4.5, 6, 9, and 12 months post-transplantation and at 6-month intervals thereafter. This study was performed in accordance with the modified Helsinki Declaration, and the protocol was approved by our ethical review boards before study initiation. Informed consent was obtained from all donors and recipients.

### Donors

Donor selection and HLA typing were performed as described previously [2, 3, 6, 18]. Donor-recipient pair was identified as HLA-identical or haploidentical matched according to the familial spectrum of genetics analysis.

### Chemotherapy prior to Allo-SCT

For the induction of CR, the patients received chemotherapy in accordance with the national ALL protocols, which included vincristine, daunorubicin, cyclophosphamide (Cy), l-asparaginase, and prednisone (VDCLP); vincristine, daunorubicin, cyclophosphamide, and prednisone (VDCP); vincristine, daunorubicin, l-asparaginase, and prednisone (VDLP); or vincristine, daunorubicin, and prednisone (VDP). Consolidation chemotherapy regimens included Cy, doxorubicin, vincristine, and dexamethasone (Hyper-CVAD [A]); methotrexate (MTX) and cytosine arabinoside (Ara-c) (Hyper-CVAD [B]); MTX and l-asparaginase; or Cy, Ara-c, and mercaptopurine (CAM), which were given in turn. Patients who did not achieve CR after induction received re-induction chemotherapy, which included VDCP; VDCLP; Ara-C, mitoxantrone, and etoposide (MAE); MTX and l-asparaginase; or Hyper-CVAD (B). Patients received re-induction chemotherapy according to doctor experience and patient intention. Prophylaxis for central nervous system leukemia consisted of intrathecal chemotherapy with at least six doses of MTX, Ara-c, and dexamethasone during induction chemotherapy and consolidation chemotherapy. The two study groups did not differ in the inductions they received (*P* = 0.52).

### Transplant protocol

Patients were treated with a myeloablative conditioning regimen according to a previous study by our group [3]. The conditioning therapies for the HID group were as follows: cytarabine (4 g/m^2^/d) intravenously on days –10 to –9; busulfan (3.2 mg/kg/d) intravenously on days –8 to –6; cyclophosphamide (1.8 g/m^2^/d), intravenously on days –5 to –4; Me-CCNU (250 mg/m^2^/d), orally once on day –3; and ATG (thymoglobulin, 2.5 mg/kg/d, Sang Stat, Lyon, France) intravenously on days –5 to –2. Patients in the MSD cohort received hydroxycarbamide (80 mg/kg) orally on day –10 and a lower dose of cytarabine (2 g/m^2^/d) on day –9, but otherwise, an identical regimen to the HID patients without ATG was employed. Acute graft-versus-host disease **(**GVHD**)** prevention and treatment were performed according to our previous study [3, 6, 19]. On the basis of bone marrow allogeneic graft CD4:CD8 ratios, patients in the HIDT cohort were categorized as low GVHD risk or high GVHD risk [19]. Patients at high GVHD risk in the HIDT cohort received low-dose corticosteroid prophylaxis [19]. In addition, two doses of 14.5 mg/kg Cy was given on days 3 and 4 after HSCT from maternal donors during the trial period [20].

### Cytomegalovirus and Epstein-Barr virus monitoring and prevention

Cytomegalovirus (CMV) and Epstein-Barr virus (EBV) levels were monitored and infections treated as described previously [3, 6, 19].

### Detection of MRD by multiparameter flow cytometry

Eight-color multiparameter flow cytometry (MFC) was performed in all patients as a routine clinical test with the sensitivity of 10^−5^ on bone marrow aspirate samples that were obtained as part of the baseline assessment before SCT, as well as 1, 2, 3, 4.5, 6, 9, and 12 months post-transplantation and at 6-month intervals thereafter.

A panel of eight antibody combinations that recognize cCD3, mCD3, CD2, CD5, CD7, CD10, CD19, CD20, CD34, CD38, CD45, CD58, CD99, CD123, and cTDT was used for MRD detection, and 0.2–1 million events per tube were acquired on a FACS Cant II. Isotype control monoclonal antibodies were used. MRD positivity was considered when a cluster of more than 20 cells with leukemia-associated immunophenotypes (LAIPs) and side scatter characteristics, identified in all plots of interest and carrying at least two LAIP markers identified at diagnosis, were observed. For those without LAIP markers at diagnosis, MRD was identified as a cell population deviating from the normal patterns of antigen expression seen on specific cell lineages at specific stages of maturation compared to either normal or regenerating marrow [21]. A lower limit of detection of 0.001% was targeted. When abnormal cells were identified, the cells were quantified as a percentage of the total CD45 positive white cell events. Any level of MRD was considered positive. The standardized assays and quality controls were performed according to previous reports [21]. The results of the MFC assessments of MRD were made available to the transplant teams. The two study groups did not differ in the proportion of patients with pre-HSCT MRDpos (29% vs. 27%, *P* = 0.73, Fig. 1).

### Interventions for MRD after transplantation

To prevent relapse, interferon-α (IFNα) was used as described in our protocol [22]. During the study period (from July 2014), PH-negative ALL patients who were MRD-positive 60 days post-transplantation was planned to receive subcutaneous IFNα-2b (3 million units) 2–3 times per week. IFNα-2b was continued for 6 months in the absence of disease progression or unacceptable toxicity. Before July 2014, most patients received preemptive G-CSF-mobilized DLI for post-HSCT MRDpos and our previous study showed comparable efficacy for IFNα-2b or DLI [22]. Thus, according to patient preference, preemptive G-CSF-mobilized DLI was also allowed in patients with post-HSCT MRDpos when donor lymphocytes were available if patients had no active GVHD [23]. Short-term immunosuppressive agents were used to prevent GVHD after DLI. The details of preemptive DLI were published previously [23, 24]. Tyrosine kinase inhibitor was preemptively administered to patients with BCR/ABL [25, 26]. The two study groups did not differ in the proportion of IFN use for the post-HSCT MRDpos intervention (67% vs. 68%, Table 1). The treatment of GVHD following IFN or DLI included methylprednisolone, prednisone, and CsA, among others.

**Table 1:** Patient and donor characteristics

Characteristics	Haplo-SCT group	MSDT group	*P* value
Number of patients	169	39	
Median age (range), years	24 (3–58)	35 (9–60)	0.001
Male sex, *n* (%)	107 (63.3%)	21 (53.8%)	0.273
Diagnosis, *n*			
B-ALL			0.142
Ph positive	49 (29.0%)	10 (25.6%)	
Ph negative	120 (55.0%)	29 (69.2%)	
T-ALL	27 (16.0%)	2 (5.1%)	
Disease status			0.328
CR1	131 (77.5%)	33 (84.6%)	
≥ CR2	38 (22.5%)	6 (15.4%)	
Median level of pre-transplant MRD (range)^#^	0.07% (0.001–6.01%)	0.05% (0.001–3.02%)	0.581
Median time from diagnosis to transplant (months, range)	6.5(3–72)	6.0(3–192)	0.803
Donor-recipient sex matched grafts, *n* (%)			0.314
Male-male	76 (45.0%)	13 (33.3%)	
Male-female	44 (26.0%)	10 (25.6%)	
Female-male	31 (18.3%)	8 (20.5%)	
Female-female	18 (10.7%)	8 (20.5%)	
Donor-recipient relationship, *n* (%)			NA
Father-child	80 (47.3%)	0	
Mother-child	12 (7.1%)	0	
Sibling-sibling	48 (28.4%)	39 (100%)	
Child-parent	24 (14.2%)	0	
Other	5 (3.0%)	0	
ABO matched grafts, *n* (%)			0.414
Matched	98 (58.0%)	19 (48.7%)	
Major mismatch	30 (17.8%)	8 (20.5%)	
Minor mismatch	33 (19.5%)	11 (28.2%)	
Bi-directional mismatch	8 (4.7%)	1 (2.6%)	
Cell compositions in grafts, mean (range)			
Infused nuclear cells, 10^8^/kg	8.16 (5.53–15.67)	8.14 (5.32–13.14)	0.711
Infused CD34^+^ cells, 10^6^/kg	2.24 (0.82–8.07)	2.45 (0.79–6.39)	0.111
Intervention for positive MRD post-HSCT among all patients, *n* (%)	39 (21%)	16 (41%)	0.027
Intervention among positive MRD post-HSCT patients, *n* (%)	39/45 (87%)	16/17(94%)	0.662
Intervention methods among positive MRD post-HSCT patients, *n* (%)			0.833
Interferon-α	29 (64%)	11 (65%)	
Donor lymphocyte infusion	6 (13%)	3 (18%)	
Targeted drug	4 (9%)	2 (12%)	
No intervention	6 (13%)	1 (6%)	

The percentages of total patients either in Haplo-SCT group or MSDT group

*Haplo-SCT* haploidentical stem cell transplantation, *MSDT* human leukocyte antigen-matched sibling donor transplantation, *ALL* acute lymphoblastic leukemia, *Ph* Philadelphia-chromosome, *CR* complete remission, *MRD* minimal (measurable) residual disease, *MA* myeloablative conditioning regimen, *NS* no significance, *DLI* donor lymphocyte infusions

^#^Indicate the percentages of MRD in total nuclear cells of bone marrow detected by multiparameter flow cytometry

### Definitions and evaluation

Engraftment, post-HSCT MRDpos, NRM, relapse, LFS, and overall survival (OS) were defined as described previously [3, 19]. Bacteremia was defined as the isolation of a bacterial pathogen from at least 1 blood culture. For coagulase-negative staphylococci and common skin contaminants, at least 2 sets of positive blood cultures were required. Invasive fungal infection (IFI) was evaluated according to the revised European Organization for Research and Treatment of Cancer/Invasive Fungal Infections Cooperative Group and the National Institute of Allergy and Infectious Diseases Mycoses Study Group (EORTC/MSG) 2008 criteria, with only proven and probable cases included. Acute GVHD was defined and graded based on the pattern and severity of organ involvement [19]. Chronic GVHD was defined and graded according to the National Institute of Health criteria [19]. Relapse was defined based on histological criteria [3, 19]. GVHD-free, relapse-free survival (GRFS) events were defined as grade III-IV aGVHD, chronic GVHD (cGVHD) requiring systemic immunosuppressive treatment, leukemia relapse, or death from any cause during follow-up after allo-HSCT.

### End-points

The primary study end-point was LFS. Secondary end-points were the engraftment rate, the incidence of acute GVHD grades II–IV and chronic GVHD, and the cumulative incidence of MRD after transplantation, relapse, NRM, OS, and GRFS. To determine whether there was any difference in LFS between MSDT and HIDT, the cumulative incidence approach was used with a one-sided confidence interval (CI) for the difference in the Kaplan-Meier estimate of the 3-year LFS. With a planned sample size of 39 MSDT patients and 169 HIDT patients, 80% power can be achieved against the hypothesis of a 20% absolute increase in LFS after HIDT (60%) from 35% of patients with pre-HSCT MRDpos leukemia free survived after MSDT at a significance level of *P* = 0.05 in the Student one-tailed *t* test [21, 27].

### Statistical analysis

The two groups were compared by the χ^2^ statistic for categorical variables and the Mann–Whitney test for continuous variables. Cumulative incidence curves were used in a competing risk setting, with relapse treated as a competing event to calculate NRM probabilities, and with death from any cause as a competing risk for GVHD, engraftment, post-HSCT MRDpos, and relapse. Time to GVHD was defined as the time from transplantation to the onset of GVHD of any grade. The probabilities of LFS and OS were estimated by the Kaplan–Meier method. All variables in Table 1 were included in the univariate analysis. Cox proportional hazards regression models were used to evaluate the relative risk of subjects undergoing transplantation by forcing the main interest variable (HID vs. MSD, using MSD as the reference group) into the model. The Fine and Gray model was used for analysis of endpoints involving competing risks. Backward elimination with a criterion of *P* < 0.10 for retention was used to select a final model. The following variables were analyzed: age at transplantation, diagnosis (PH positive B-ALL vs. PH negative B-ALL vs. T-ALL), disease status (CR2 vs. CR1), time from diagnosis to HSCT, donor-recipient sex match (female-male vs. others), donor source (MSD vs. HID), pre-HSCTMRD level, post-HSCTMRD status (neg vs. pos), and acute and chronic GVHD. Unless otherwise specified, *P* values were based on two-sided tests. Alpha was set to 0.05. Most analyses were performed in SPSS 16.0 (Mathsoft, Seattle, WA, USA).

## Results

### Study population

A total of 745 ALL patients who achieved CR after chemotherapy were enrolled in this study (Fig. 1). Twenty of these patients were excluded due to receiving matched unrelated donor transplantation, and 517 patients were excluded due to achieving CR with pre-HSCT MRDneg. Finally, 208 cases with pre-HSCT MRDpos were genetically randomized into the HIDT (*n* = 169) and MSDT groups (*n* = 39).

Patients, disease, and donor characteristics are summarized in Table 1. One-hundred twenty patients were Ph− B-ALL (58%), 59Ph+ B-ALL (28%), and 29 (14%) were T-ALL. Recipients of HIDT and MSDT were comparable concerning gender, time from diagnosis to HSCT, disease subtype and status, and pre-HSCT MRD level (Table 1). However, patients in the MSDT group were older than those in the HIDT group; also, as mentioned above, each HIDT patient received ATG while MSDT patient did not, and low-dose corticosteroid prophylaxis was given to 89 patients in the HIDT cohort.

### Engraftment, GVHD, and infection

All patients achieved sustained, full donor chimerism. The 100-day cumulative incidence of platelet engraftment in the HIDT group was significantly lower than that in the MSDT group (95%, 95% CI, 92–98% vs. 100%, *P* < 0.001, Table 2). Multivariate analysis (MVA) showed that CD34 cell infused was the only significant factor associated with both neutrophil and platelet engraftment (Table 3).

**Table 2:** Transplant outcomes between patients who underwent Haplo-SCT and those who received MSDT

Parameter	Haplo-SCT group (*n* = 169)	MSDT group (*n* = 39)	*P* value
Median time of neutrophil engraftment (range)	13 days (10–25 days)	15 days (9–22 days)	0.016
Platelet engraftment at day 100 post-transplantation	95% (95% CI, 92–98%)	100%	< 0.001
CMV reactivation at day 100 post-transplantation	68% (95% CI, 61–75%)	18% (95% CI, 6–30%)	< 0.001
EBV reactivation at day 100 post-transplantation	15% (95% CI, 10–21%)	0	0.011
Grades II–IV acute GVHD	21% (95% CI, 17–27%)	23% (95% CI, 10–36%)	0.884
Total chronic GVHD	44% (95% CI, 36–52%)	48% (95% CI, 31–65%)	0.850
Moderate-to-severe chronic GVHD	18% (95% CI, 10–26%)	27% (95% CI, 10–44%)	0.192
Cumulative incidence of positive MRD after transplantation	26% (95% CI, 19–33%)	44% (95% CI, 28–60%)	0.043
Three-year probability of relapse	23% (95% CI, 17–29%)	47% (95% CI, 31–63%)	0.006
Three-year probability of NRM	11% (95% CI, 6–16%)	10% (95% CI, 1–19%)	0.845
Three-year probability of LFS	65% (95% CI, 58–72%)	43% (95% CI, 27–59%)	0.023
Three-year probability of OS	68% (95% CI, 61–75%)	46% (95% CI, 30–62%)	0.039
Three-year probability of GRFS	54% (95% CI, 46–62%)	36% (95% CI, 21–51%)	0.055

*Haplo-SCT* haploidentical stem cell transplantation, *MSDT* human leukocyte antigen-matched sibling donor transplantation, *CI* confidence interval, *GVHD* graft-versus-host disease, *MRD* measurable residual disease, *NRM* non-relapse mortality, *LFS* leukemia-free survival, *OS* overall survival

**Table 3:** Uni- and multivariate analysis of factors associated with transplantation outcomes (*n* = 208)

Covariate	Univariate analysis			Multivariate analysis		
	HR	95% CI	*P* value	HR	95% CI	*P* value
**Neutrophil engraftment**						
CD34 cell infused (less vs. higher than median)	0.776	0.589–1.021	0.070	0.749	0.567–0.988	0.041
Transplant modality (Haplo-SCT vs. MSDT)	1.275	0.899–1.809	0.173			
**Platelet engraftment**						
CD34 cell infused (less vs. higher than median)	0.648	0.490–0.857	0.002	0.671	0.506–0.889	0.006
Transplant modality (Haplo-SCT vs. MSDT)	0.388	0.269–0.561	< 0.001	0.401	0.227–0.581	< 0.001
**Acute GVHD grades II–IV**						
Disease status (≥ CR2 vs. CR1)	2.464	1.333–4.557	0.004	2.468	1.330–4.578	0.004
Transplant modality (Haplo-SCT vs. MSDT)	1.081	0.502–2.325	0.842			
**Total chronic GVHD**						
Time from diagnosis to transplant	1.011	1.000–1.022	0.053	1.011	1.000–1.022	0.050
Transplant modality (Haplo-SCT vs. MSDT)	0.949	0.548–1.641	0.850			
**MRD positive after transplantation**						
Disease status (≥ CR2 vs. CR1)	1.690	0.943–3.031	0.078			
Levels of pre-transplantation MRD	1.243	1.014–1.523	0.036	1.281	1.043–1.572	0.014
Transplant modality (Haplo-SCT vs. MSDT)	0.526	0.301–0.921	0.024	0.492	0.280–0.866	0.018
**Relapse**						
Disease status (≥ CR2 vs. CR1)	2.356	1.343–4.134	0.003	2.528	1.357–4.707	0.003
Diagnosis			0.037			0.002
T-ALL	2.757	1.235–6.157	0.013	4.356	1.814–10.460	0.001
PH negative B-ALL	1.379	0.710–2.678	0.343	1.368	0.690–2.713	0.370
PH positive B-ALL	1.0			1.0		
Levels of pre-transplantation MRD	2.204	1.267–3.835	0.005	1.320	1.060–1.642	0.013
Chronic GVHD (yes vs. no)	0.475	0.263–0.859	0.014	0.337	0.181–0.628	0.001
Post-transplantation MRD	2.168	1.283–3.664	0.004	2.149	1.253–3.685	0.005
Transplant modality (Haplo-SCT vs. MSDT)	0.465	0.265–0.815	0.008	0.360	0.197–0.655	0.001
**Non-relapse mortality**						
Platelet engraftment (Yes vs. no)	0.038	0.015–0.097	< 0.001	0.048	0.018–0.122	< 0.001
Grades II–IV acute GVHD	3.382	1.481–7.723	0.004	2.573	1.102–6.008	0.029
Transplant modality (Haplo-SCT vs. MSDT)	1.095	0.372–3.218	0.869	0.663	0.213–2.061	0.478
**Leukemia-free survival**						
Disease status (≥ CR2 *vs.* CR1)	2.015	1.238–3.278	0.005	1.789	1.059–3.024	0.030
Diagnosis			0.013			0.021
T-ALL	2.653	1.351–5.208	0.005	2.696	1.308–5.558	0.007
PH negative B-ALL	1.357	0.776–2.371	0.284	1.375	0.765–2.472	0.287
PH positive B-ALL	1.0			1.0		
Levels of pre-transplantation MRD	1.358	1.160–1.590	< 0.001	1.747	1.011–3.021	0.046
Platelet engraftment (yes vs. no)	0.130	0.062–0.275	< 0.001	0.153	0.067–0.352	< 0.001
Grades II–IV acute GVHD	1.991	1.232–3.218	0.005	1.727	1.036–2.879	0.036
Chronic GVHD (yes vs. no)	0.474	0.288–0.781	0.003	0.476	0.282–0.803	0.005
Post-transplantation MRD	1.500	0.955–2.357	0.078	1.707	1.059–2.752	0.028
Transplant modality (Haplo-SCT vs. MSDT)	0.570	0.349–0.933	0.025	0.425	0.252–0.718	0.001
**Overall survival**						
Disease status (≥ CR2 vs. CR1)	2.311	1.404–3.806	0.001	2.238	1.321–3.790	0.003
Diagnosis			0.015			0.005
T-ALL	2.938	1.414–6.105	0.004	3.602	1.670–7.770	0.001
PH negative B-ALL	1.631	0.890–2.987	0.113	1.938	1.044–3.596	0.036
PH positive B-ALL	1.0			1.0		
Levels of pre-transplantation MRD	1.312	1.107–1.555	0.002	1.202	0.975–1.482	0.085
Platelet engraftment (yes vs. no)	0.077	0.036–0.169	< 0.001	0.083	0.034–0.199	< 0.001
Grades II–IV acute GVHD	2.555	1.559–4.185	< 0.001	2.426	1.442–4.082	0.001
Chronic GVHD (yes vs. no)	0.482	0.292–0.794	0.004	0.469	0.269–0.820	0.008
Post-transplantation MRD	1.438	0.915–2.259	0.116	1.649	0.990–2.746	0.055
Transplant modality (Haplo-SCT vs. MSDT)	0.584	0.348–0.980	0.042	0.395	0.225–0.695	0.001

All variables were first included in the univariate analysis; only variables with *P*<0.1 and the forced variable (transplant modality) were included in the Cox proportional hazards model with time-dependent variables

*HR* hazard ratio, *CI* confidence interval, *MRD* minimal residual disease, *CR* complete remission, *Haplo-SCT* haploidentical stem cell transplantation, *MSDT* human leukocyte antigen-matched sibling donor transplantation, *ALL* acute lymphoblastic leukemia, *GVHD* graft-versus-host disease

The cumulative, 100-day incidence of acute GVHD grades II–IV and grades III–IV in the HIDT group was similar to that of the MSDT group (21%, 95% CI, 17–27% vs. 23%, 95% CI, 10–36%; *P* = 0.884; and 6%, 95% CI, 3–9% vs. 5%, 95% CI, 0–12%; *P* = 0.838). In addition, the 3-year cumulative incidence of total chronic GVHD and moderate to severe chronic GVHD was comparable between the HIDT and MSDT groups (44%, 95% CI, 36–52% vs. 48%, 95% CI, 31–65%; *P* = 0.850; and 18%, 95% CI, 10–26% vs. 27%, 95% CI, 10–44%; *P* = 0.192). MVA indicated that disease status was correlated with acute GVHD while time from diagnosis to HSCT affecting chronic GVHD (Table 3).

The 100-day cumulative incidence of CMV or EBV reactivation after engraftment in the HIDT group was significantly higher than that in the MSDT group (68%, 95% CI, 61–75% vs. 18%, 95% CI, 6–30%; *P* < 0.001; 15%, 95% CI, 10–21% vs. 0; *P* = 0.011; Table 2). The 100-day cumulative incidence of bacteremia or IFI after engraftment in the HIDT group was comparable to that in the MSDT group (4%, 95% CI, 1–7% vs. 3%, 95% CI, 0–8%; *P* = 0.784; 8%, 95% CI, 4–12% vs. 3%, 95% CI, 0–8%; *P* = 0.342).

### Non-relapse mortality and causes of death

**Fig. 2:**
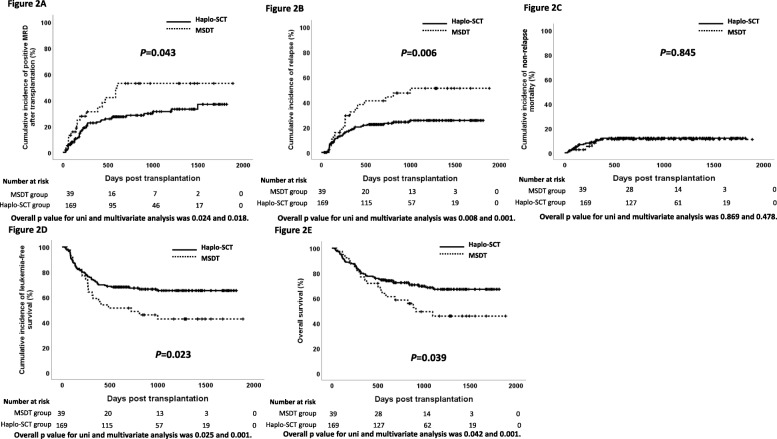
Outcome of allogeneic stem cell transplantations in two cohorts after a median follow-up of 820 days. **a** Cumulative incidence of positive measurable/minimal residual disease after transplantation (overall *P* value for uni- and multivariate analysis was 0.024 and 0.018). **b** Cumulative incidence of leukemia relapse (overall *P* value for uni- and multivariate analysis was 0.008 and 0.001). **c** Non-relapse mortality (overall *P* value for uni- and multivariate analysis was 0.869 and 0.478). **d** Leukemia-free survival (overall *P* value for uni- and multivariate analysis was 0.025 and 0.001). **e** Overall survival (overall *P* value for uni- and multivariate analysis was 0.042 and 0.001). Abbreviations: Haplo-SCT, haploidentical stem cell transplantation; MSDT, human leukocyte antigen-matched sibling donor transplantation; MRD, measurable residual disease

The 3-year cumulative incidence of NRM was comparable between the HIDT and MSDT groups (11%, 95% CI, 6–16% vs. 10%, 95% CI, 1–19%; *P* = 0.845; Table 2 and Fig. 2c). MVA demonstrated that platelet engraftment and acute GVHD affected NRM (Table 3). Causes of death are shown in Table 4. Infection was the major cause of NRM.

**Table 4:** Primary cause of death among patients that underwent allogeneic stem cell transplantation

Cause of death	Haplo-SCT group (*n* = 51)	MSDT group (*n* = 20)
**Relapse**	32 (62.7%)	16 (80.0%)
**Infection**	12 (23.5%)	2 (10.0%)
**Graft failure**	3 (5.9%)	0 (0%)
**GVHD**	1 (2.0%)	2 (10.0%)
**Others**	3 (5.9%)	0 (0%)

Values represent the number (percentage) of deaths among the total number of patients in each of the two cohorts

*Haplo-SCT* haploidentical stem cell transplantation, *MSDT* human leukocyte antigen-matched sibling donor transplantation, *GVHD* graft-versus-host disease, *VOD* hepatic veno-occlusive disease

### Post-HSCT MRDpos, interventions, and relapse

Until the last follow-up, 62 patients with post-HSCT MRDpos were observed, 45 in the HIDT group and 17 in the MSDT group. The cumulative incidence of post-HSCT MRDpos was 26% (95% CI, 19–33%) and 44% (95% CI, 28–60%; *P* = 0.043, Table 2 and Fig. 2a). MVA showed that an HID was a beneficial factor, while higher level of pre-HSCT MRDpos was a risk factor for post-HSCT MRDpos (Table 3).

Details of preemptive interventions for post-HSCT MRDpos are described in Table 1. Seven patients, including six in the HIDT group and one in the MSDT group, received no intervention because these cases relapsed within 2 weeks after the detection of MRD post-transplantation (*n* = 6) or had active GVHD (*n* = 1, Table 1). As mentioned above, we found no significant differences in the different preemptive methods between these two groups, with IFN being the most frequently used modality (*P* = 0.83, Table 1). After preemptive interventions, 21 patients (21/55, 38%) eventually experienced hematological relapse, including 12/39 HIDT cases (31%) and 9/16 MSDT cases (56%; *P* = 0.12). In the HIDT or MSDT cohort, 9 of 29 patients (31%) and 7 of 11 (64%) receiving IFNα (*P* = 0.08), 3 of 6 (50%) and 2 of 3 (66%) receiving preemptive DLI, and none of the 6 (0%) receiving tyrosine kinase inhibitor (TKI) relapsed, respectively.

The 3-year cumulative incidence of post-HSCT MRD or hematological relapse, whichever occurred first, was 38% (95% CI, 31–45%) and 64% (95% CI, 50–78%) in the HIDT and MSDT groups, respectively (*P* = 0.006). Compared to those receiving HIDT, patients who underwent MSDT had a higher 3-year CIR (47%, 95% CI, 31–63% vs. 23%, 95% CI, 17–29%; *P* = 0.006; Table 2 and Fig. 2b). The therapies of relapse included chemotherapy followed by therapeutic DLI (*n* = 21), TKI followed by DLI (*n* = 4), chimeric antigen receptor T-cell immunotherapy (CART) cell infusion (*n* = 3), and the others received chemotherapy (*n* = 10) or TKI alone (*n* = 2) or no therapy (*n* = 16). MVA showed that an HID and occurrence of chronic GVHD were beneficial factors, while T-ALL, more than CR1, higher level of pre-HSCT MRDpos, and post-HSCT MRDpos were risk factors for relapse (Table 3).

### LFS, OS, and GRFS

Compared to those receiving HIDT, patients who underwent MSDT had a lower 3-year LFS (43%, 95% CI, 27–59% vs. 65%, 95% CI, 58–72%; *P* = 0.023; Fig. 2d) and OS (46%, 95% CI, 30–62% vs. 68%, 95% CI, 61–75%; *P* = 0.039; Fig. 2e) and a trend of lower GRFS (36%, 95% CI, 21–51% vs. 54%, 95% CI, 46–52%; *P* = 0.055; Table 2). MVA showed that an HID, platelet engraftment, and occurrence of chronic GVHD were beneficial factors, while T-ALL, more than CR1, higher level of pre-HSCT MRDpos, occurrence of acute GVHD, and post-HSCT MRDpos were risk factors for LFS and OS (Table 3).

### Subgroup analysis for patients with sensitivity at 0.01% in the bone marrow for pre-HSCT MRDpos

With the aim that the study results can be compared with literature, we did analysis for patients with sensitivity at 0.01% in the bone marrow for pre-HSCT MRDpos (*n* = 128) including 24 MSDT and 104 HIDT. The 3-year CIR, LFS, and OS was 46% (95% CI, 24–68%) vs. 29% (95% CI, 20–38%; *P* = 0.159), 37% (95% CI, 17–57%) vs. 55% (95% CI, 45–65%; *P* = 0.175) and 43% (95% CI, 23–63%) vs. 59% (95% CI, 49–69%; *P* = 0.270) for patients who underwent MSDT or HIDT, respectively, whereas NRM rate was similar (17%, 95% CI, 0–34% vs. 15%, 95% CI, 8–22%; *P* = 0.782).

### Outcomes for patients with pre-HSCT MRDneg

During the study period, patients with pre-HSCT MRDneg (*n* = 517) were also randomized genetically to choose MSD (*n* = 92) or HID (*n* = 425, Fig. 1). Patients, disease, and donor characteristics are summarized in Table S1. Compared to those receiving HIDT, patients who underwent MSDT had comparable 3-year CIR (16%, 95% CI, 8–24% vs. 15%, 95% CI, 11–19%; *P* = 0.776), LFS (72%, 95% CI, 63–81% vs. 68%, 95% CI, 64–72%; *P* = 0.463), OS (73%, 95% CI, 64–82% vs. 70%, 95% CI, 66–74%; *P* = 0.528), and NRM (12%, 95% CI, 5–19% vs. 16%, 95% CI, 12–20%; *P* = 0.274, Table S2 and Figure S1).

### Outcomes for PH+ ALL patients

During the study period, PH+ ALL patients (*n* = 194) were also randomized genetically to choose. There are 124 Ph+ ALL cases with pre-HSCT MRDpos determined by real-time polymerase chain reaction (RT-PCR) in the 725 ALL patients. In this subgroup, we found that the CIR between HIDT (*n* = 48) and MSDT (*n* = 18) was 18% (95% CI, 10–37%) and 12% (95% CI, 5–19%), respectively (*P* = 0.450). The NRM, LFS, and OS between these HIDT and MSDT groups were also comparable (data not shown). There are fifty-five Ph+ ALL cases with pre-HSCT MRDpos determined by RT-PCR in the 208 patients with pre-HSCT MRDpos detected by 8-color MFC. In this subgroup, we found that the CIR between HIDT (*n* = 48) and MSDT (*n* = 7) was 17% (95% CI, 6–28%) and 43% (95% CI, 5–80%), respectively (*P* = 0.106). The NRM, LFS, and OS between these HIDT and MSDT groups were also comparable (data not shown).

## Discussion

The criteria for selecting the most appropriate transplant donor remain a topic of ongoing debate [4, 5, 8, 9, 11, 12, 14, 15]. This prospective, genetically randomized study provided the most robust evidence thus far that HIDT is superior to MSDT, potentially due to stronger GVL effects in certain patients. As opposed to most retrospective comparative studies between HIDT and MSDT, or a few prospective cohorts with limited statistical power [5, 8–11, 13]. The strengths of this analysis include a relatively large number of homogenous patients using consistent supportive care algorithms, conditioning regimens, and stem cell sources. Such genetically randomized studies are one means of providing guidance regarding a change in the traditional donor hierarchy of MSD being first choice in certain circumstances [14, 15].

In theory, choosing a donor with greater HLA disparity from the recipient could mitigate the relapse risk induced by a greater allo-immune GVL effect [5, 16, 28]. However, apart from donor type, many factors can influence GVL [2, 22, 23, 25], including disease type and remission status before HSCT, patient age, conditioning regimen, GVHD prophylaxis, number of T cells infused, presence of GVHD, use of immunotherapy and targeted drugs, and other factors. Because of the profound effect of MRD on transplant outcomes [21, 27, 29], this situation was chosen for close examination in the scenario of evaluating donor selection. The current study demonstrated that, for ALL patients with pre-HSCT MRDpos, HIDT can achieve lower CIR and better survival than MSDT. Moreover, we observed that, compared to patients undergoing MSDT, patients who underwent HID had a lower incidence of post-HSCT MRDpos and a lower proportion required preemptive therapy. All patients in the two study groups were treated with similar conditioning regimens without in vitro T-cell depletion. The one disparity in the GVHD prophylaxis schedule was that all HIDT recipients received ATG, which was not used for MSDT recipients [3, 19]. The requirement for additional immunosuppression in the HIDT protocol is an integral aspect of the current standards of GVHD prophylaxis [6, 19]. Furthermore, the use of ATG to facilitate engraftment and prevent GVHD without influencing relapse may not weaken the GVL effect [30]. Also, low-dose corticosteroid prophylaxis given to high GVHD risk HIDT patients did not influence relapse (data not shown), which confirmed our previous results [19]. Another unbalanced feature between the two groups is patient age. Due to the one child policy in China, half of our HIDs were parents [2, 3, 6]; thus, an age difference existed between the two study groups (Table 1). Though younger patients are prone to biologically less aggressive leukemia [31], our study populations were all transplanted in CR with pre-HSCT MRDpos, and age was not a significant factor affecting CIR in the multivariate analysis. Therefore, the lower incidence of post-HSCT MRDpos and lower CIR in the HIDT group cannot be explained exclusively by any of the confounding factors discussed. Our data, together with other studies on AML or Hodgkin’s lymphoma [8, 9], offer the most compelling evidence that choosing a HID over MSD has a favorable anti-leukemia effect [32]. However, the biological and immunological mechanism of donor choice based on GVL needs to be explored further. Apart from donor source, disease type and status also affected CIR, with T-ALL being worse than B-ALL and more than CR1 being inferior to CR1 as expected.

As disease control can be improved with greater use of immunomodulatory or targeted approaches [22, 23], we described preemptive interventions for MRD post-transplantation. Among patients with post-HSCT MRDpos, both the proportion and methods of preemptive interventions were similar between the two groups, with IFN most frequently used, followed by DLI [22, 23]. HIDT tended to have a lower relapse rate than MSDT after preemptive intervention for MRD post-transplantation (31% vs. 56%, *P* = 0.12) or in subgroup analysis after IFN (31% vs. 64%, *P* = 0.08). These data confirmed the predictive role of MRD on prognosis [21, 29] and the effectiveness of preemptive therapies in both transplant modalities, but they also provide further evidence that HIDT offers an advantage over MSDT in terms of a better response to preemptive interventions for MRD post-transplantation [22, 23].

Weighing the likelihood of relapse versus GVHD and non-relapse mortality could guide donor selection [2, 4, 14, 15]. The similar rate of acute GVHD between the two groups was somewhat contradictory to our previous comparative studies with higher grades II–IV acute GVHD in HIDT than MSDT [3, 6]. Our risk stratification-directed, low-dose corticosteroid prophylaxis for GVHD in HIDT and lower proportion of female donors (29% vs. 41%) in the HIDT group may contribute to the comparable GVHD incidence between the two cohorts [2, 19]. Instead, disease status and time from diagnosis to HSCT affected acute or chronic GVHD. The equivalent NRM was in accordance with our previous reports of acute leukemia patients transplanted in CR [3, 10]. Thus, the HLA-antigen mismatch with HIDs contributes to greater allo-immunity against the tumor without affecting the allo-immunity against the host, partly due to advancements in GVHD prevention and infection control [2, 6, 19]. Thus, the tension between relapse and NRM translated to superior survival after HIDT [4]. Apart from donor source, T-ALL, more than CR1, acute GVHD had detrimental effect on survival while platelet engraftment and chronic GVHD had protective effect on survival. In addition, patients with pre-HSCT MRDneg had a higher LFS than those with pre-HSCT MRDpos in MSDT settings (*P* = 0.004), but LFS was comparable in HIDT settings for patients with pre-HSCT MRDneg versus pre-HSCT MRDpos (Figure S1), which indicated that, in accordance with our previous results of AML patients [10], HIDT could obscure the negative effect of pre-HSCT MRDpos in ALL patients while MSDT could not.

Regarding health-related quality of life (HRQoL), our previous retrospective study showed that the HRQoL of patients receiving HIDT is comparable to that of patients receiving MSDT [33], and chronic GVHD severity strongly correlates with negative impacts on patients’ HRQoL [34]. Although the current study does not include HRQoL analysis, cGVHD incidence and severity was comparable between the 2 groups and GRFS was higher in HIDT cohort. Further prospective studies investigating HRQoL are needed to evaluate if HID should replace MSD in some situation.

## Conclusions

In conclusion, this prospective genetically randomized study is powered to detect that HIDT beats MSDT in regard to favorable anti-leukemia activity for ALL patients with pre-HSCT MRDpos. The current study paves the way to determine that HIDs should be the preferred choice regardless of available MSDs in a subgroup population. Our findings warrant further investigation and could inform decision-making and the development of donor-selection algorithms [2, 4, 14, 15, 18, 35]. More multi-center, prospective trials and mechanism studies are necessary to evaluate donor selection in regard to the anti-leukemia effect.

## Supplementary information

**Additional file 1: Figure S1.** Outcome of allogeneic stem cell transplantations in four groups classified according to pre-transplantation MRD and transplant modalities (n=725). (A) cumulative incidence of leukemia relapse, (B) non-relapse mortality, (C) leukemia-free survival, and (D) overall survival. Abbreviations: Haplo-SCT=haploidentical stem cell transplantation; MSDT=human leukocyte antigen-matched sibling donor transplantation; MRD=measurable residual disease.

**Additional file 2: Table S1.** Patient and donor characteristics (n=517)*. **Table S2.** Transplant outcomes between patients with negative pre-transplantation MRD who underwent Haplo-SCT and those who received MSDT (n=517).

## Data Availability

The datasets used and/or analyzed during the current study are available from the corresponding author on reasonable request.

## Abbreviations

ALL: Acute lymphoblastic leukemia
AML: Acute myeloid leukemia
ATG: Anti-thymocyte globulin
Ara-c: Cytosine arabinoside
CAM: Cyclophosphamide, cytosinearabinoside, mercaptopurine
CART: Chimeric antigen receptor T-cell immunotherapy
cGVHD: Chronic graft-versus-host disease
CI: Confidence interval
CIR: Cumulative incidence of relapse
CR: Complete remission
CMV: Cytomegalovirus
Cy: Cyclophosphamide
DLI: Donor lymphocyte infusion
EBMT: European Society for Blood and Marrow Transplantation
EBV: Epstein-Barr virus
EORTC/MSG: European Organization for Research and Treatment of Cancer/Invasive Fungal Infections Cooperative Group and the National Institute of Allergy and Infectious Diseases Mycoses Study Group
GRFS: GVHD-free, relapse-free survival
GVHD: Graft-versus-host disease
GVL: Graft-versus-leukemia
HIDs: Haploidentical donors
HIDT: Haploidentical donor transplantation
HLA: Human leukocyte antigen
HRQoL: Health-related quality of life
HSCT: Hemopoietic stem cell transplantation
Hyper-CVAD [A]: Cyclophosphamide, doxorubicin, vincristine, and dexamethasone
Hyper-CVAD [B]: Methotrexate and cytosine arabinoside
IFI: Invasive fungal infection
IFNα: Interferon-α
LAIPs: leukemia-associated immunophenotypes
LFS: Leukemia-free survival
MAE: Cytosinearabinoside, mitoxantrone, and etoposide
MFC: Multiparameter flow cytometry
MRD: Minimal residual disease
MSDs: Matching sibling donors
MSDT: Matched sibling donor transplantation
MTX: Methotrexate
MVA: Multivariate analysis
NRM: Non-relapse mortality
OS: Overall survival
PH: Philadelphia chromosome
post-HSCT MRDpos: Post-transplantation minimal residual disease positivity
pre-HSCT MRDpos: Pre-transplantation minimal residual disease positivity
RT-PCR: Real-time polymerase chain reaction
SCT: Stem cell transplantation
TKI: Tyrosine kinase inhibitor
URD: Unrelated donor
VDCLP: Vincristine, daunorubicin, cyclophosphamide, L-asparaginase, and prednisone
VDCP: Vincristine, daunorubicin, cyclophosphamide, and prednisone
VDLP: Vincristine, daunorubicin, L-asparaginase, and prednisone
VDP: Vincristine, daunorubicin, and prednisone
