# Supplementary material for: Haploidentical donor is preferred over matched sibling donor for pre-transplantation MRD positive ALL: a phase 3 genetically randomized study
Source: J Hematol Oncol. 2020 Mar 30;13:27. doi: 10.1186/s13045-020-00860-y (PMC7106867; doi:10.1186/s13045-020-00860-y)
Supplement: Supplementary file 1 — Additional file 1: Figure S1. Outcome of allogeneic stem cell transplantations in four groups classified according to pre-transplantation MRD and transplant modalities (n=725). (A) cumulative incidence of leukemia relapse, (B) non-relapse mortality, (C) leukemia-free survival, and (D) overall survival. Abbreviations: Haplo-SCT=haploidentical stem cell transplantation; MSDT=human leukocyte antigen-matched sibling donor transplantation; MRD=measurable residual disease. [file 13045_2020_860_MOESM1_ESM.ppt]

## Slide 1
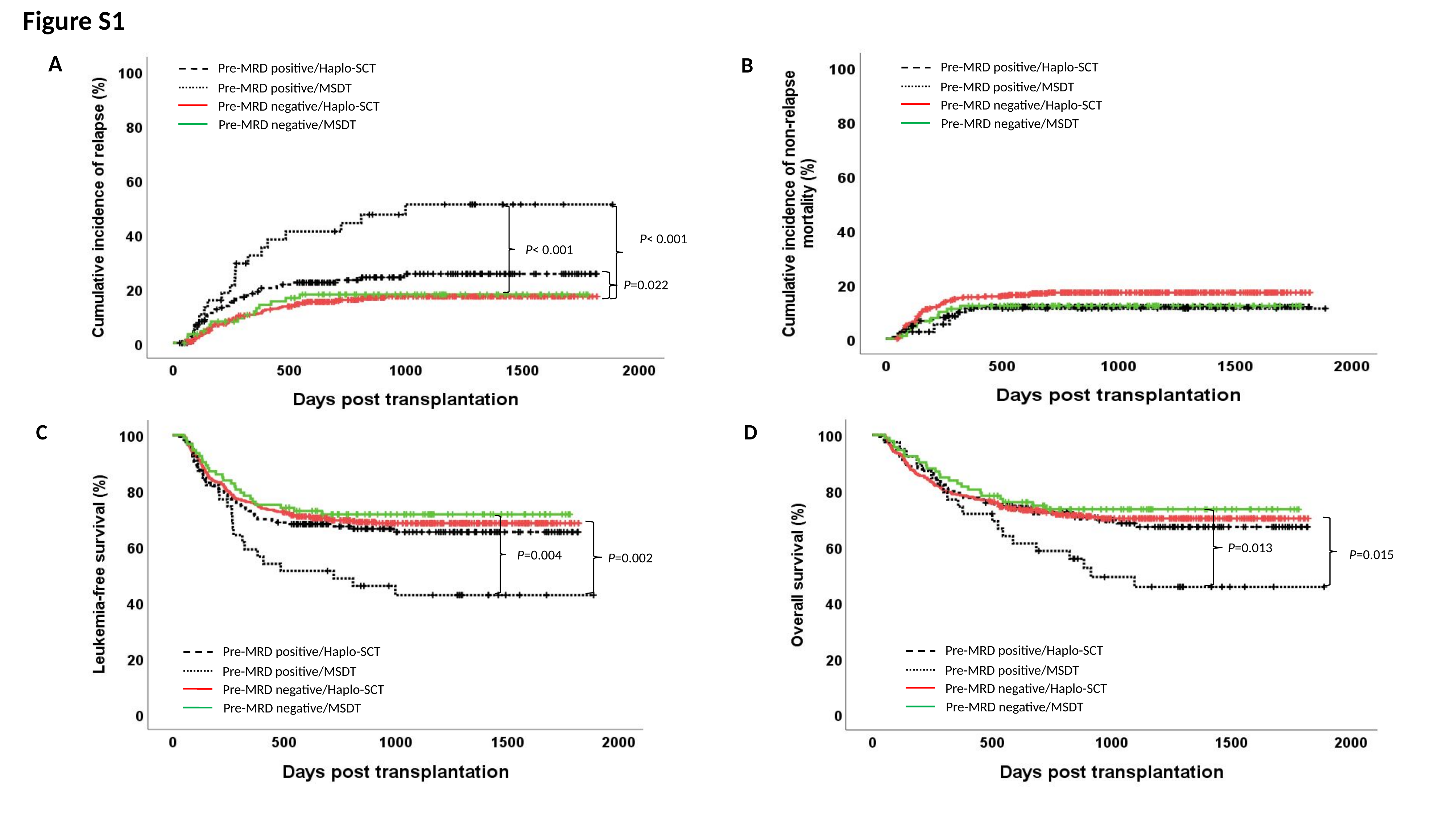

Figure S1
A
B
Pre-MRD positive/Haplo-SCT
Pre-MRD positive/MSDT
Pre-MRD negative/Haplo-SCT
Pre-MRD negative/MSDT
Pre-MRD positive/Haplo-SCT
Pre-MRD positive/MSDT
Pre-MRD negative/Haplo-SCT
Pre-MRD negative/MSDT
P< 0.001
P< 0.001
P=0.022
C
D
P=0.013
P=0.015
P=0.004
P=0.002
Pre-MRD positive/Haplo-SCT
Pre-MRD positive/MSDT
Pre-MRD negative/Haplo-SCT
Pre-MRD negative/MSDT
Pre-MRD positive/Haplo-SCT
Pre-MRD positive/MSDT
Pre-MRD negative/Haplo-SCT
Pre-MRD negative/MSDT
