# Supplementary material for: Haploidentical donor is preferred over matched sibling donor for pre-transplantation MRD positive ALL: a phase 3 genetically randomized study
Source: J Hematol Oncol. 2020 Mar 30;13:27. doi: 10.1186/s13045-020-00860-y (PMC7106867; doi:10.1186/s13045-020-00860-y)
Supplement: Supplementary file 2 — Additional file 2: Table S1. Patient and donor characteristics (n=517)*. Table S2. Transplant outcomes between patients with negative pre-transplantation MRD who underwent Haplo-SCT and those who received MSDT (n=517). [file 13045_2020_860_MOESM2_ESM.docx]

**Table S1.** Patient and donor characteristics (n=517)*

| characteristics | MSDT group | Haplo-SCT group | *P* value |
| --- | --- | --- | --- |
| Number of patients | 92 | 425 |  |
| Median age (range),years | 31 (5-60) | 24 (2-59) | <0.001 |
| Male sex, n (%) | 57 (62.0%) | 263 (61.9%) | 0.989 |
| Diagnosis, n |  |  |  |
| B-ALL | 66 (71.7%) | 318 (74.8%) | 0.539 |
| T-ALL | 26 (28.3%) | 107 (25.2%) |  |
| Patient subgroup, n (%) |  |  | 0.495 |
| Ph positive | 27 (29.3%) | 110 (25.9%) |  |
| Disease status |  |  | 0.113 |
| CR1 | 85 (92.4%) | 367 (86.4%) |  |
| ≥ CR2 | 7 (7.6%) | 58 (13.6%) |  |
| Median level of pre-transplant MRD (range)^#^ | 0 | 0 | NS |
| Median Time from diagnosis to transplant (months, range) | 6.25(3.5-41) | 6.0(2.5-125) | 0.648 |
| Conditioning regimen, n (%) |  |  |  |
| MA | 92 (100%) | 425 (100%) | NS |
| Donor-recipient sex matched grafts, n (%) |  |  | < 0.001 |
| Male-male | 24 (26.1%) | 212 (49.9%) |  |
| Male-female | 16 (17.4%) | 120 (28.2%) |  |
| Female-male | 34 (37.0%) | 58 (13.6%) |  |
| Female-female | 18 (19.6%) | 35 (8.2%) |  |
| Donor-recipient relationship, n (%) |  |  | - |
| Father-child | 0 | 244 (57.4%) |  |
| Mother-child | 0 | 24 (5.6%) |  |
| Sibling-sibling | 92 (100%) | 107 (25.2%) |  |
| Child-parent | 0 | 40 (9.4%) |  |
| Other | 0 | 10 (2.4%) |  |
| ABO matched grafts, n (%) |  |  | 0.414 |
| Matched | 54 (58.7%) | 234 (55.1%) |  |
| Major mismatch | 15 (16.3%) | 92 (21.6%) |  |
| Minor mismatch | 17 (18.5%) | 86 (20.2%) |  |
| Bi-directional mismatch | 6 (6.5%) | 13 (3.1%) |  |
| Cell compositions in grafts, mean (range) |  |  |  |
| Infused nuclear cells, 10^8^/kg | 7.89 (5.11-13.81) | 8.41 (2.75-15.43) | < 0.01 |
| Infused CD34^+^ cells, 10^6^/kg | 2.29 (0.52-8.51) | 2.50 (0.47-12.44) | 0.100 |

**Abbreviations:** Haplo-SCT=haploidentical stem cell transplantation; MSDT=human leukocyte antigen-matched sibling donor transplantation; ALL=acute lymphoblastic leukemia; Ph=philadelphia-chromosome; CR=complete remission; MRD=minimal (measurable) residual disease; MA=myeloablative conditioning regimen; NS=no significance; DLI=Donor lymphocyte infusions

* indicate the percentages of total patients either in haplo-SCT group or MSDT group

^#^ indicate the percentages of MRD in total nuclear cells of bone marrow detected by multiparameter flow cytometry

**Table S2.** Transplant outcomes between patients with negative pre-transplantation MRD who underwent Haplo-SCT and those who received MSDT (n=517)

| Parameter | MSDT group (n=92) | Haplo-SCT group (n=425) | *P* value |
| --- | --- | --- | --- |
| Median time of neutrophil engraftment, days (range) | 15 (10-24) | 13 (9-26) | <0.001 |
| Platelet engraftment at day 100 post-transplantation | 96% (95% CI, 92%-100%) | 92% (95% CI, 89%-95%) | 0.029 |
| Grades II-IV acute GVHD | 13% (95% CI, 6%-20%) | 29% (95% CI, 25%-33%) | 0.002 |
| Total chronic GVHD | 56% (95% CI, 45%-67%) | 50% (95% CI, 45%-55%) | 0.194 |
| Three-year probability of relapse | 16% (95% CI, 8%-24%) | 15% (95% CI, 11%-19%) | 0.776 |
| Three-year probability of NRM | 12% (95% CI, 5%-19%) | 17% (95% CI, 13%-21%) | 0.274 |
| Three-year probability of LFS | 72% (95% CI, 63%-81%) | 68% (95% CI, 64%-72%) | 0.463 |
| Three-year probability of OS | 73% (95% CI, 64%-82%) | 70% (95% CI, 66%-74%) | 0.528 |

**Abbreviations:** Haplo-SCT=haploidentical stem cell transplantation; MSDT=human leukocyte antigen-matched sibling donor transplantation; CI=confidence interval; GVHD=graft-versus-host disease; MRD=measurable residual disease; NRM=non-relapse mortality; LFS=leukemia-free survival; OS=overall survival
